# Short term visual outcomes of a new trifocal intraocular lens

**DOI:** 10.1186/s12886-017-0462-y

**Published:** 2017-05-17

**Authors:** Jorge L. García-Pérez, Juan Gros-Otero, Celia Sánchez-Ramos, Vanesa Blázquez, Inés Contreras

**Affiliations:** 1 Clínica Rementería, c/Almagro 36 Entreplanta Dcha, 28015 Madrid, Spain; 2 Department of Optics, Faculty of Optics and Optometry, Complutense University, Madrid, Spain

**Keywords:** Trifocal intraocular lens, Multifocal intraocular lens, Cataract surgery

## Abstract

**Background:**

Today, patients often expect to achieve spectacle independance after cataract surgery. New trifocal intraocular lenses have been developed to try and fullfill this demand. The purpose of this study is to report the short-term visual outcomes of a new trifocal intraocular lens (AcrySof PanOptix™).

**Methods:**

Consecutive adult patients undergoing cataract surgery with bilateral implantation of the study intraocular lens in a private practice clinic were considered for inclusion. Exclusion criteria were the presence of other ocular pathologies or preoperative astigmatism >1.5 diopters (D). Patients with intraoperative complications were excluded from analysis. One month after surgery patients underwent: monocular defocus curve; monocular and binocular uncorrected visual acuity in photopic and mesopic conditions, for far (4 m), intermediate (60 cm) and near (33 cm) distances and binocular contrast sensitivity. Patients completed a visual satisfaction questionnaire between 9 and 12 months after surgery.

**Results:**

One hundred and sixteen eyes of fifty-eight patients receiving bilateral implantation of the study intraocular lens were analysed. Mean binocular uncorrected visual acuity in photopic conditions was 0.03 LogMAR for far, 0.12 for intermediate and 0.02 for near distances. All patients achieved a binocular uncorrected visual acuity better than 0.3 LogMAR (20/40 Snellen equivalent) for distance and near vision and 94.8% of patients for intermediate vision. Mesopic binocular uncorrected visual acuity values were similar to photopic values. The monocular defocus curves showed that the best visual acuity was reached at a vergence of 0.00D. Visual acuity dropped slightly at −1.00D and peaked again at −2.00D. Visual acuities better than 0.2 LogMAR were maintained between −2.50D and +0.50D. Contrast sensitivity was high and similar in photopic and mesopic conditions. As regards patient-evaluated outcomes, only 2 patients (3.4%) were fairly dissatisfied with their sight after surgery. Three patients (5.1%) reported the need for spectacle correction for certain activities. All other patients (94.8%) reported never using spectacle correction.

**Conclusions:**

The PanOptix trifocal IOL provides good short-term visual outcomes, with good intermediate performance and excellent patient-reported satisfaction. The similar values achieved in mesopic and photopic conditions in binocular uncorrected visual acuity and contrast sensitivity suggest low pupillary dependence for light distribution.

**Trial registration number:**

ISRCTN60143265, retrospectively registered on the 24th of April 2017.

## Background

Intraocular lens (IOL) design is continuously evolving in order to improve visual outcomes, increase patient satisfaction and achieve spectacle-independence after cataract surgery. Diffractive bifocal IOLs were designed with concentric rings which create a near and far focus; pupillary changes help to adjust light distribution between both focuses to improve visual function [1]. A drawback of bifocal IOLs is that intermediate performance is often below the requirements for activities such as computer use or correct dashboard perception while driving [2, 3]. Trifocal technology has been developed to create a true intermediate focus to overcome these difficulties. Initial reports on the visual outcomes of the FineVision® (Physiol, Liège, Belgium) and AT LISA tri839MP® (Carl Zeiss Meditec, Jena, Germany) trifocal IOLs are encouraging [4–12].

The new AcrySof PanOptix® trifocal IOL (Alcon Research, Fort Worth, TX, USA) has been developed to improve light transmission and distribution between the three focuses. Its design aims to decrease pupillary dependence for excellent performance and to improve intermediate vision. To the best of our knowledge, so far there have been no reports on daily practice clinical outcomes with this new trifocal IOL. The purpose of this study was to evaluate clinical outcomes in patients with bilateral PanOptix lens implantation.

## Methods

This study was a prospective case series evaluating visual function in patients scheduled for bilateral implantation of the studied IOL. The study adhered to the tenets of the declaration of Helsinki and was approved by the ethics committee of the Hospital Clínico San Carlos, Madrid. Inclusion criteria were patients over 18 years old candidates for bilateral cataract surgery. Exclusion criteria were the presence of any ocular pathology which could compromise visual recovery, preoperative astigmatism higher than 1.5 Diopters (D) on corneal topography or abnormal iris.

Candidates for cataract surgery underwent an extensive evaluation including: best-corrected visual acuity (BCVA), anterior segment biomicroscopic evaluation, intraocular pressure measurement, corneal topography (Pentacam HR model 70,900, Oculus, Germany), specular biomicroscopy (CEM-530, NIDEK CO, LDT, Japan), dilated fundus examination, optical coherence tomography examination of the macula and optic nerve (Cirrus HD-OCT 5000, Carl Zeiss Meditec AG, Germany) and IOL calculation with the IOL Master 700 (Carl Zeiss Meditec AG, Germany). After these explorations and an in-depth discussion of the characteristics of monofocal and multifocal lens, the ophthalmologist recommended the intraocular lens best suited to the patient. If the recommended lens was the Panoptix IOL, the patient was considered for inclusion in the study. The purpose of the study was explained to patients with none of the exclusion criteria and patients agreeing to participate signed an informed written consent.

Surgery was scheduled first for the eye with the worst visual acuity. The other eye underwent surgery between one and 10 days later. Patients were seen on the day after the intervention and between 30 and 40 days (1-month visit) after the second procedure. Patients with any intraoperative or postoperative complications were excluded from analysis. At the 1-month visit, all explorations performed preoperatively were repeated. In addition, the following specific explorations of the study were performed at the 1-month visit. Patients underwent: monocular defocus curve; mono- and binocular uncorrected visual acuity in photopic and mesopic conditions, for far, intermediate and near distances; subjective refraction and binocular contrast sensitivity in photopic and mesopic conditions.

For photopic visual acuity measurements, room luminance was 85 cd [cd]/m^2^. Monocular and binocular uncorrected distance visual acuity were measured using a 22″ LED liquid crystal display system (CC-100 HW 5.0 Series, Topcon) that can display ETDRS charts at 4 m. Monocular and binocular uncorrected near visual acuity were measured using the Logarithmic Visual Acuity Chart 2000 New ETDRS (Precision Vision, Lasalle, IL) at 33 cm. Intermediate visual acuity was also assessed both mono- and binocularly, at 60 cm.

Subjective refraction was performed with the ETDRS chart at 4 m. The defocus curve was then performed monocularly with the patients observing the ETDRS chart through lenses starting at −5.00 D and increasing in 0.50 D steps to +3.00 D. Binocular contrast sensitivity was measured at spatial frequencies of 3, 6, 12, and 18 cycles per degree (cpd) using the functional acuity contrast test (Test SV-1000) of the CC-100 HW 5.0 Series system.

After dark adaptation (10 min in the testing room under mesopic conditions), mesopic monocular and binocular uncorrected distance visual acuity were measured with the room luminance set to 3 cd/m^2^. Mono- and binocular near and intermediate visual acuity were also measured in mesopic conditions, as well as binocular contrast sensitivity. Absolute log10 contrast sensitivity (log10 CS) values were obtained and the mean values and standard deviations were calculated.

Between 9 and 12 months after surgery the patients were contacted and asked to fulfill the Catquest 9-SF questionnaire, which has been recently validated in a Spanish population [13]. Since this questionnaire does not specifically ask about certain issues that are important when evaluating the outcomes of trifocal IOLs, five other questions were added to the questionnaire (Table 1).

**Table 1:** Questions added to the Catquest 9-SF questionnaire with options provided

1. Do you use spectacle correction…
For near distance (for reading or sewing)?
Never
Sometimes
Always
For intermediate distance (using computers or cooking)?
Never
Sometimes
Always
For far distances (driving, cinema)?
Never
Sometimes
Always
2. Do you see halos with low illumination (during the night or if there is little light)?
Never
Occasionally
Often
Always
3. Do light sources provoke glare with low illumination (such as driving at night)?
Never
Occasionally
Often
Always
4. Do you have difficulties when driving at night?
Never
Occasionally
Often
Always
5. Would you undergo the same surgery again?
Yes
No

### Intraocular lens

**Fig. 1:**
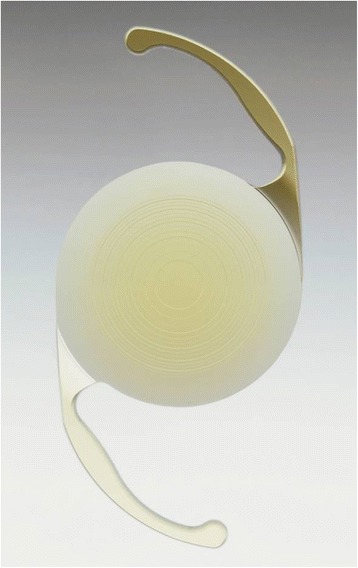
The PanOptix intraocular lens. It is a single-piece, aspheric, non-apodized diffractive lens with a 6.0-mm biconvex optic, a central trifocal zone of 4.5 mm, an overall diameter of 13.0 mm, and 0-degree haptic angulation

The PanOptix IOL is a single-piece, aspheric, non-apodized diffractive IOL with a 6.0-mm biconvex optic, an overall diameter of 13.0 mm, and 0-degree haptic angulation. It has a central trifocal zone of 4.5 mm, designed to reduce pupillary dependence (Fig. 1). For a pupil diameter of 3 mm, it transmits 88% of incident light with an asymmetric distribution of 50% to the distance focus and 25% for the intermediate and near foci. It has an addition of +3.25 D for the near focus and a + 2.17 D addition for the intermediate focus at the IOL plane.

### Statistics

Statistics were performed with the SPSS Advanced Statistical 20.0 software (SPSS, Inc., Chicago, IL). Quantitative data are provided as ranges, means and standard deviations (SD). The Student t-test was used to compare normally distributed data as confirmed using the Kolmogorov-Smirnov test, and non-parametric tests for non-normally distributed data. Significance was set at a *p* ≤ 0.05. *P* values provided are two-tailed.

## Results

Sixty patients were initially included in the study. One patient was excluded from analysis because at the 1 month visit he had postsurgical macular edema in one eye. Another patient was excluded because there was a posterior capsular tear during surgery, although a posterior capsular rexis was performed and the IOL was finally implanted in the bag. Therefore, fifty-eight patients were included in the analysis, with 45 women (77.6%) and 13 men (22.4%). Mean age was 69.3 years (standard deviation [SD] 9.79 years), ranging between 43 and 85 years.

### Visual acuity and refractive status

**Fig. 2:**
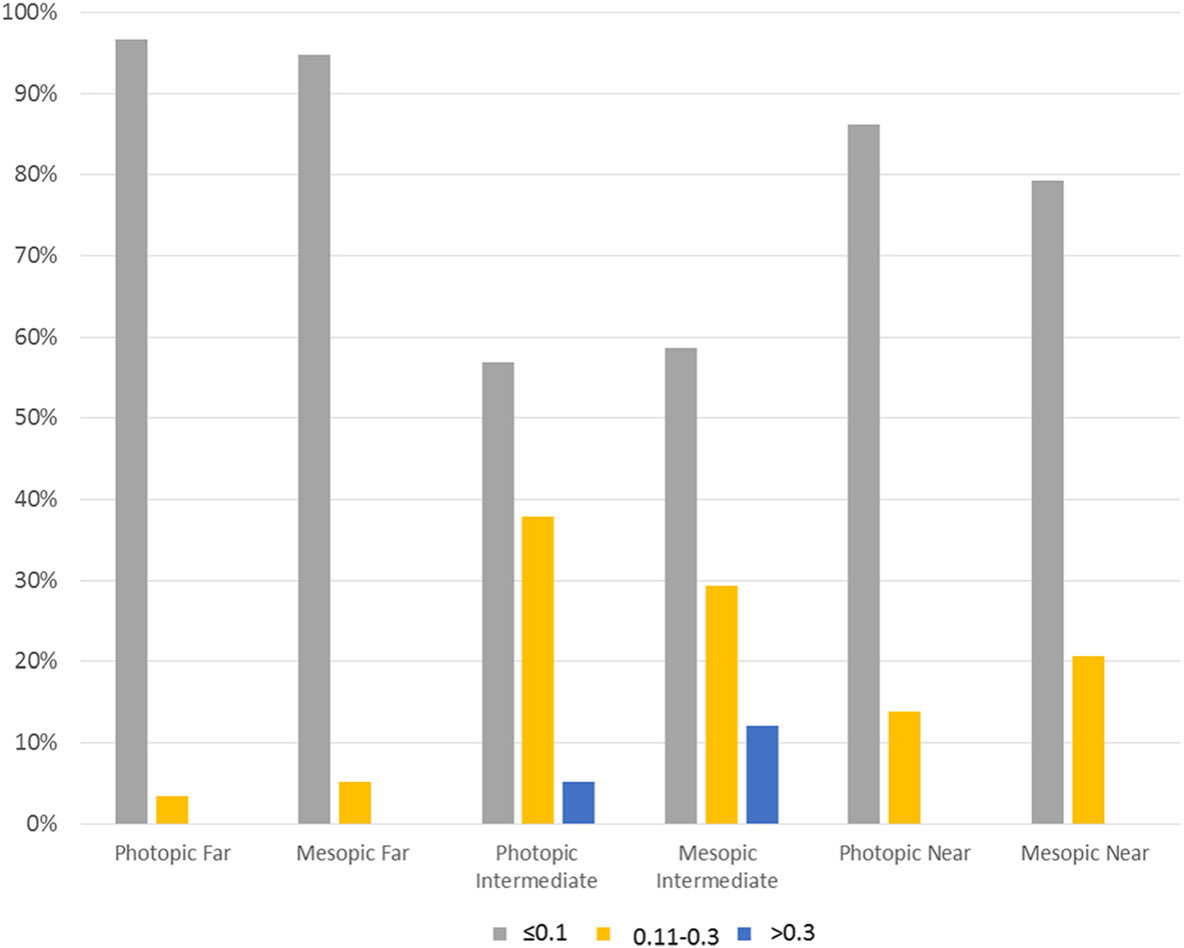
LogMAR visual acuity distribution for uncorrected binocular visual acuity, for photopic (85 cd/m2) and mesopic conditions (3 cd/m2), for far (4 m) intermediate (60 cm) and near (33 cm) distances

Table 2 records mono- and binocular uncorrected visual acuity. There were no significant differences between the values for photopic and mesopic conditions (*p* > 0.05 for all comparisons). One month after surgery, all patients achieved an uncorrected photopic binocular visual acuity of 0.3 LogMAR (Snellen equivalent 20/40) or better for far and near distances, with 96.6% achieving a distance and 86.2% a near acuity of 0.1 LogMAR (Snellen equivalent 20/25) or better (Fig. 2). For intermediate distance, 56.9% of patients reached an uncorrected binocular acuity better than 0.1 and 37.9% were between 0.3 and 0.1. Only 5.2% didn’t reach an intermediate acuity better than 0.3. Mesopic visual acuities were similar to photopic values, with slightly lower percentages of patients reaching 0.1.

**Table 2:** Full-contrast logMAR uncorrected visual acuity. Data are provided as the mean (standard deviation) and range

		Right eye	Left eye	Binocular
Photopic (85 cd/m^2^)	Far (4 m)	0.06 (0.090) 0.30 to −0.1	0.06 (0.078) 0.26 to −0.1	0.03 (0.046) 0.14 to −0.16
	Intermediate (60 cm)	0.20 (0.182) 0.60 to −0.10	0.18 (0.145) 0.60 to −0.06	0.12 (0.143) 0.50 to −0.18
	Near (33 cm)	0.08 (0.116) 0.40 to −0.12	0.07 (0.109) 0.32 to −0.16	0.02 (0.099) 0.30 to −0.18
Mesopic (3 cd/m^2^)	Far (4 m)	0.06 (0.746) 0.26 to 0	0.05 (0.070) 0.28 to 0	0.03 (0.048) 0.24 to 0
	Intermediate (60 cm)	0.21 (0.169) 0.64 to − 0.1	0.19 (0.152) 0.64 to − 0.1	0.12 (0.148) 0.5 to − 0.16
	Near (33 cm)	0.09 (0.119) 0.40 to − 0.10	0.08 (0.124) 0.44 to − 0.16	0.03 (0.108) 0.26 to − 0.18

*cd/m* ^*2*^ candelas per square meter, *m* meters, *cm* centimeters

Mean postoperative spherical equivalent was −0.10 D ± 0.26 (range − 0.87 to +0.75 D). Postoperative spherical equivalent was between −0.50 and +0.50 D in 94.8% of eyes, with 4.3% (5 eyes) between −1.00 and −0.50 D and 0.9% (1 eye) between +0.50 and +1.00 D.

### Defocus curve

**Fig. 3:**
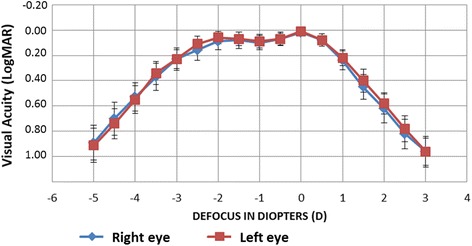
Monocular distance-corrected defocus curve given in logMAR 1 month after surgery

Figure 3 shows the through-focus corrected monocular logMAR visual acuity. The best visual acuity (0.02 [SD 0.06] and 0.01 [SD 0.05] for the right and left eyes) was reached at a vergence of 0.00 D, corresponding to the far focus. Visual acuity dropped slightly at −1.00 D, corresponding to the intermediate focus and then peaked again at −2.00 D (near focus). Visual acuities of 0.2 or better were maintained between −2.50 and +0.50 D.

### Contrast sensitivity

**Fig. 4:**
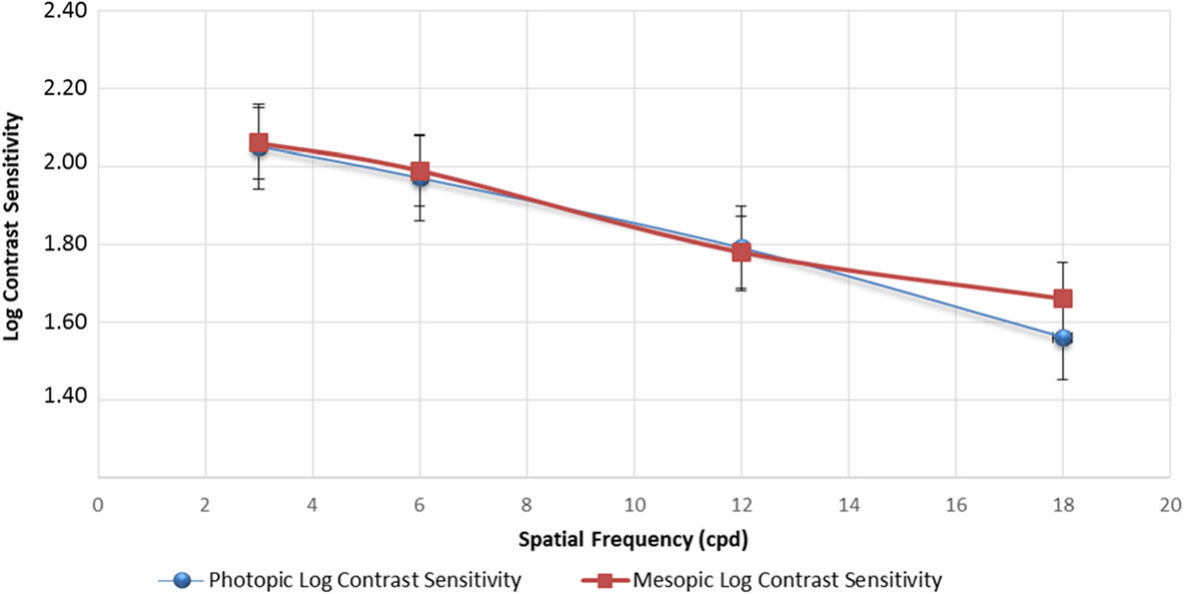
Mean binocular contrast sensitivity function in photopic (85 cd/m2) and mesopic conditions (3 cd/m2) 1 month after surgery

Figure 4 shows the mean binocular log10 CS values under photopic (85 cd/m^2^) and mesopic (3 cd/m^2^) conditions. Contrast sensitivity was similar in photopic and mesopic conditions (*p* > 0.05 for all spatial frequencies).

### Visual satisfaction questionnaire

**Fig. 5:**
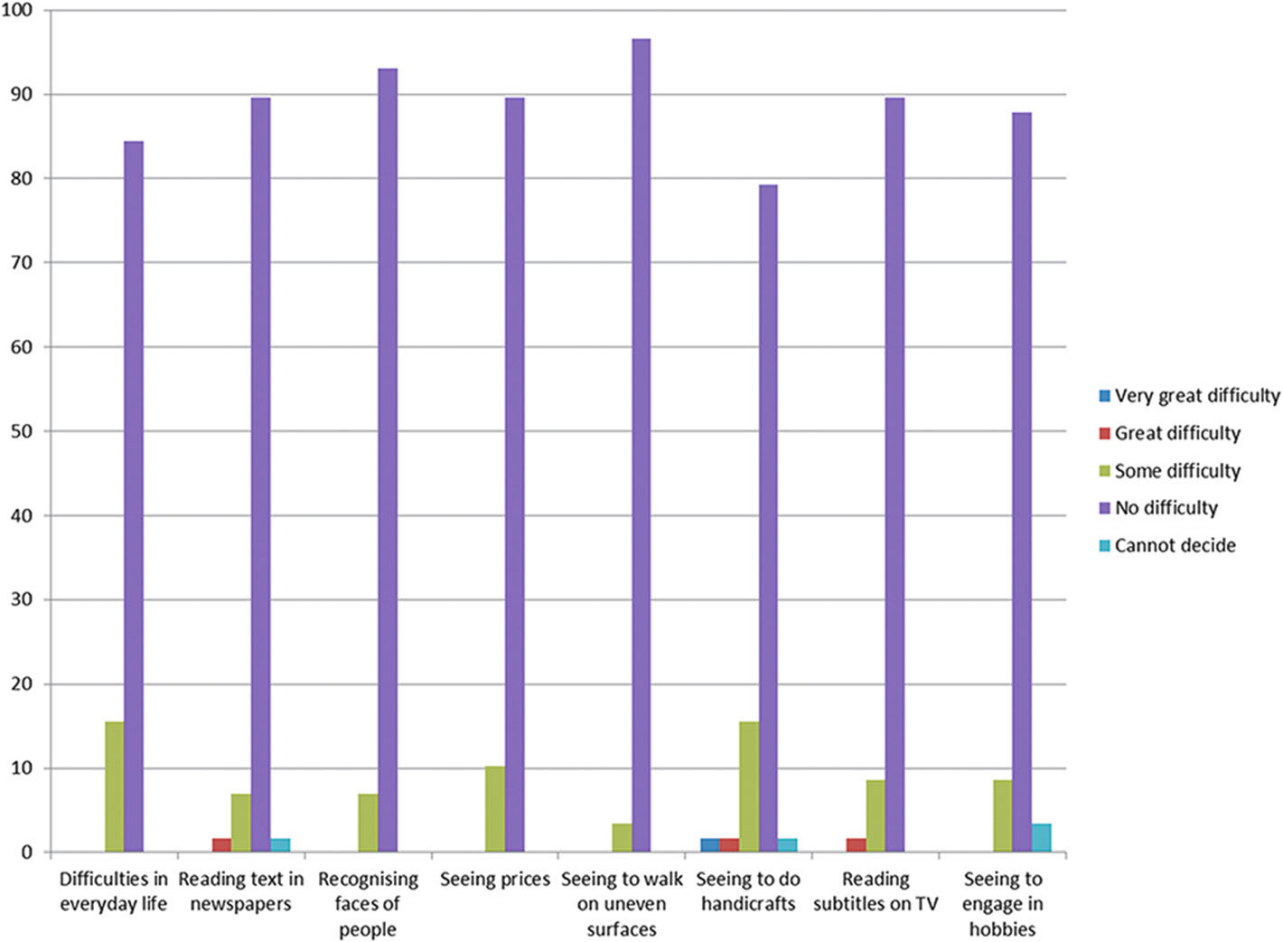
Patient’ answers to question 3 of the Catquest 9-SF questionnaire, which explores difficulties in performing different activities of daily life

On the Catquest 9-SF questionnaire, 49 patients (84.5%) reported their vision caused them no difficulties in their daily lives. Nine patients (15.5%) reported having some difficulties. As regards their current vision, 32 patients (55.2%) were very satisfied, 24 patients (41.4%) quite satisfied and 2 patients (3.4%) quite unsatisfied. Of the two unsatisfied patients, when further questioned, one of them actually complained of tearing and itching and not really of visual difficulties. Another had difficulties due to posterior capsule opacification and was scheduled for YAG capsulotomy. Figure 5 shows the answers to the other questions on the Catquest 9-SF questionnaire. More than 79% of patients reported having no difficulties in performing all tasks.

**Fig. 6:**
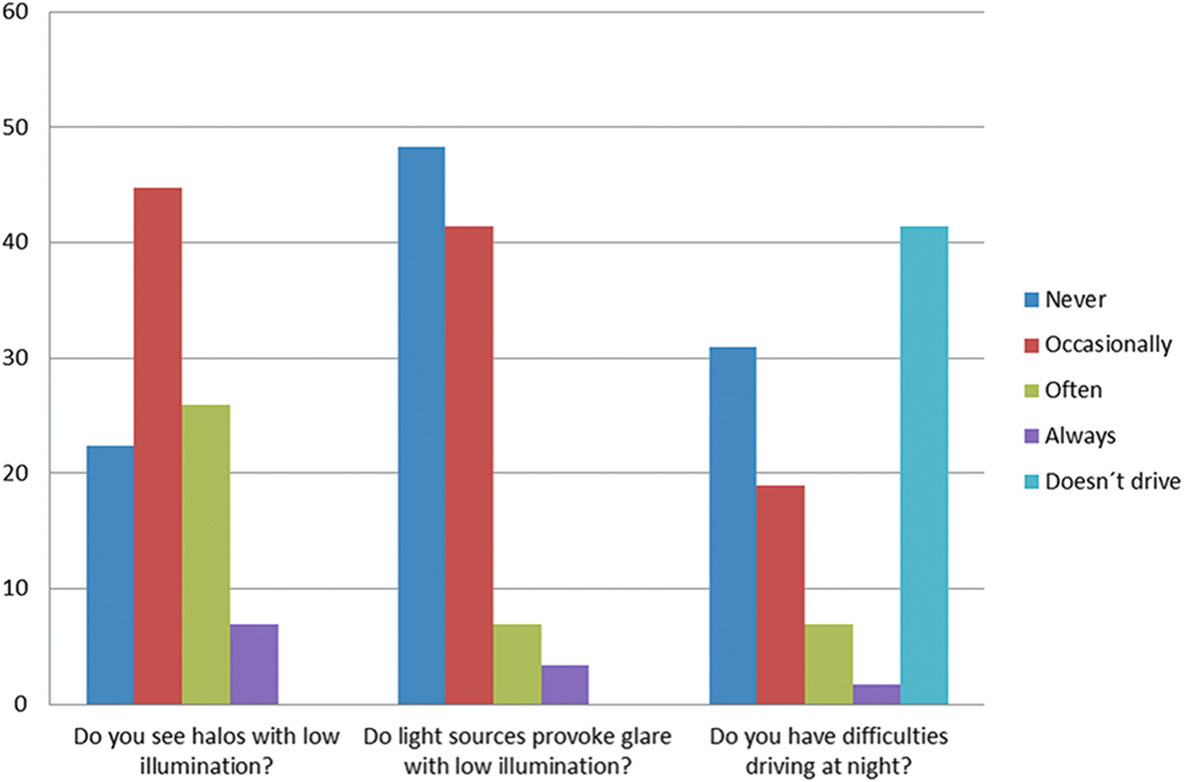
Patient’ reported incidence of halos and glare, as well as of difficulties when driving at night

As regards spectacle use, for near vision 56 patients (96.6%) never used spectacles, one patient (1.7%) sometimes and another patient (1.7%) always. Only one patient (1.7%) reported using spectacles sometimes for intermediate vision. One patient (1.7%) reported using spectacle correction always for far vision, two patients (3.4%) sometimes and 55 patients (94.8%) never. Figure 6 shows the patient’ reported incidence of halos and glare and the difficulties for driving at night. Four patients reported they wouldn’t undergo the same surgery again: three of them due to ocular surface problems (tearing, itching, red eye). The other one was the patient with posterior capsule opacification.

## Discussion

Multifocal IOLs were developed to satisfy patients’ demands to be spectacle-independent after cataract surgery. Although bifocal IOLs provided good visual function for far and near distances, intermediate performance often did not meet patients’ expectations. Trifocal technology has been developed to improve intermediate visual function, which is necessary for activities such as using laptops, smartphones and tablets, playing cards, reading price tags or focusing on the dashboard while driving. The PanOptix IOL has a novel diffractive structure which would allow a higher light utilization, transmitting 88% of light to the retina at a simulated 3.0 mm pupil size. Bench Badal image testing and modulation transfer measurements have shown that the PanOptix is equivalent to bifocal IOLs in photopic near and distance performance while providing a substantial range of intermediate foci with an optimal intermediate focus at 60 cm [14]. However, we are not aware of reports of visual outcomes of the PanOptix in daily clinical practice.

We performed this prospective observational study in order to report the early results after cataract surgery with bilateral implantation of the PanOptix. Visual performance was evaluated 1 month after surgery because previous studies have not found significant differences in visual acuity one, three and 6 months after surgery in patients receiving trifocal lens [4, 6, 7, 10, 15]. Similarly, contrast sensitivity has also been found to be stable between 1 and 12 months postoperatively [9]. As regards the distances at which intermediate and near visual acuity were evaluated, studies performed with a reading desk have reported that the preferred intermediate distance ranges between 61.50 cm and 64.20 cm [14, 16]; 60 cm was chosen because it is very similar to this preferred range and has been already used in other publications [4, 5, 15], facilitating comparison with studies performed with other IOLs. Similarly, the mean reported preferred near distance ranges between 34.6 cm and 38.70 cm [14, 16] and 33 cm was chosen as the distance to evaluate near vision as used in one other study which compared two trifocal IOLs [15].

We found that the monocular and binocular uncorrected distance, intermediate and near visual acuities 1 month after binocular implantation of the PanOptix IOL were high and similar to those described for other trifocal lens [4–6, 10, 12, 15, 17]. One of the concerns about trifocal technology is that the light distribution to create an intermediate focus might interfere with the far and near focuses and reduce visual acuity. However, the uncorrected distance and near vision achieved by our patients were similar to other studies with bifocal lens [2, 6, 18], suggesting that the addition of an intermediate focus does not interfere with the other two focuses.

Comparisons with other studies describing trifocal IOL visual outcomes are difficult because of the different characteristics of the patients included and the different methods for measuring visual acuity and contrast sensitivity employed in each study (Table 3). Binocular uncorrected distance visual acuity was similar or slightly worse in our study than reported for the AT LISA Tri, although a higher percentage of patients achieved an uncorrected binocular visual acuity better than 0.1 [4, 7, 19]. Binocular uncorrected distance visual acuity was also slightly better with the Finevision [5, 6, 15]. Intermediate visual acuity was similar for the PanOptix (0.12 LogMAR) to the values reported for 60 cm for the AT LISA Tri (between 0.11 [4] and 0.13 LogMAR [15]) and slightly worse than reported for the FineVision (between 0.06 [5] and 0.03 LogMAR [15]). Near acuity (0.02 LogMAR) was similar to that reported for the FineVision (between 0.00 [5] and 0.02 LogMAR [15] for 30 to 33 cm) and better than reported for the AT LISA (between 0.13 and 0.32 [4, 15]).

**Table 3:** Previous studies reporting visual outcomes with trifocal intraocular lens

First author *Lens studied*	Patients included Mean age (years)	Mean uncorrected binocular visual acuity (% of patients with uncorrected binocular acuity <0.1)		
		Far (4 m)	Intermediate (60 cm)	Near (33 cm)
Garcia-Perez (current study) *PanOptix*	58 patients 69.3 ± 9.8	0.03 ± 0.04 (96.6%)	0.12 ± 0.14 (56.9%)	0.02 ± 0.09 (86.2%)
Alfonso [4] *AT LISA tri 839MP*	102 patients 60.5 ± 8.5	0.03 (86.1%)	0.11 (28.7%)	0.32^a^ (0%)
Kohnen [7] *AT LISA tri 839MP*	27 patients 64 ± 7.9	- 0.06 ± 0.09	- 0.01 ± 0.10^d^	0.03 ± 0.11^c^
Kretz [19] *AT LISA tri 839MP*	50 patients 59.3 ± 7.6	0.04 (91%*)	0.04^e^ (79%*)	0.01^c^ (87%*)
Mendicute [10] *AT LISA tri 839MP*	104 patients	(89.3%)	(67.7%)^d^	(52%)^c^
Marques [15] *Finevision Micro F* *AT LISA tri 839MP*	15 patients 71 ± 7 15 patients 70 ± 5	0.02 ± 0.02 0.00 ± 0.01	0.03 ± 0.054 0.13 ± 0.424	0.02 ± 0.023 0.13 ± 0.053
Cochener [5] *Finevision*	99 patients 66.9 ± 9.1	0.01 ± 0.07	0.06 ± 0.08	0.00 ± 0.03^a^
Jonkers [6] *Finevision*	15 patients 62.6 ± 8.7	0.01 ± 0.08	0.33 ± 0.10^b^	0.11 ± 0.11^c^

*LogMAR ≤0.00

Distances at which intermediate and near visual acuities were measured that are other than described in the headings are as follows:

^a^30 cm; ^b^70 cm; ^c^40 cm; ^d^80 cm; ^e^66 cm

Defocus curves are usually performed binocularly in order to replicate real life situations. Kretz et al. showed that the effect of binocular fusion gave an average gain of one line for all distances [19]. We performed monocular defocus curves to determine the true range of focus provided by the IOL per se, without the effect of binocular summation. Defocus curves for bifocal IOLs typically show two humps, corresponding to the visual acuity peaks for the far (0.00D) and near (−2.50D) focuses, with decreased acuity for the intermediate range (from-1.00 to −2.00D) [2, 18, 20]. The monocular defocus curves for the PanOptix in our study (Fig. 3) showed two peaks, at 0 and −2.00D, but visual acuity remained excellent in between, with a LogMAR acuity ≤0.1 between +0.50 and −2.00 D. Similar curves have been described for other trifocal IOLs [5, 6, 8, 12, 17, 21–24]. Kohnen et al. also evaluated the defocus curve monocularly for the AT LISA Tri; visual acuity dropped to approximately 0.2 LogMAR for −1.50 and −2.00 D [7], a slightly worse result than we found.

Another concern about trifocal technology is whether light distribution may vary from the optimum in different luminance levels. We did not find differences in visual acuities between photopic and mesopic conditions, a fact that would support that light distribution for the PanOptix is less dependent on pupillary size. Multifocal IOL design might also lead to a reduction in contrast sensitivity, since light from the out-of-focus image reduces the sharpness of the in-focus image. However, we found that the studied IOL had very good contrast sensitivity values, with mean photopic values of 2.05, 1.97, 1.79 and 1.56 for 3, 6, 12 and 18 cpd respectively. There were no significant differences with mesopic values. Contrast sensitivity was measured binocularly, in order to better evaluate the impact of the IOL on a situation more similar to daily life and to facilitate comparisons with previous studies performed with other IOLs. Photopic contrast sensitivity values were slightly better than described for the FineVision (between 1.64 and 1.66 for 3 cpd, 1.71 and 1.77 for 6 cpd, 1.09 and 1.44 for 12 cpd and 0.62 and 0.96 [6, 22, 23]) and for the ATLISA (1.56, 1.66, 1.37 and 0.94 for 3, 6, 12 and 18 cpd respectively [23]).

As regards patients’ ability to perform daily tasks without spectacle correction, one patient reported using spectacles occasionally for all distances, one patient sometimes for far distances and one patient always for near and far distances. This last patient was an 83-year-old lady with 1.50D residual astigmatism in her right eye who did not desire to undergo further surgery to correct it. Most patients reported no or little difficulty for the activities included in the Catquest 9-SF questionnaire. Driving at night was, as expected, the most challenging activity: 15 patients (25.9%) reported having difficulties occasionally or often and 1 patient (1.7%) always. Studies performed with the FineVision IOL have reported that between 95 and 100% of patients were spectacle-free for distance and approximately 20% patients needed glasses for near distance between three and six months after surgery [5, 6]. Reports on spectacle independence with the AT LISA Tri are less consistent, with spectacle requirement for near vision ranging between 10 and 30% [7, 10, 20]. The perception of photic phenomena is almost unavoidable with multifocal IOLs. They are reported by up to 90% of patients implanted with trifocal IOLs, although most describe them as not bothersome [8, 10]. Furthermore, the perception of photic phenomena decreases with time [8, 17]. In our study, 19 patients (32.8%) reported seeing halos often or always with low illumination and 6 patients (10.3%) reported glare.

This study has several limitations. The number of patients included is relatively low and no comparison was made with other trifocal IOLs. One month is a short follow-up period. Patients might experience refractive changes with time, as well as visual acuity decreases due to posterior capsule opacification. Longer follow-up would be necessary to more precisely characterize the IOLs outcomes. It must also be taken into account that patients completed the visual satisfaction questionnaire between nine and 12 months after surgery and therefore not at the time clinical outcomes were evaluated. This is another limitation of the study, since for instance photic phenomena have been described to decrease with time and neuroadaptation might influence perceived outcomes.

In summary, the present study found that the new PanOptix trifocal IOL provided good short-term visual outcomes, with uncorrected monocular and binocular visual acuities for all distances consistent with those reported for other trifocal IOLs. The defocus curves suggest that patients will have a satisfactory range of intermediate vision. Contrast sensitivity was high, suggesting light scattering is low. The fact that there were no differences between photopic and mesopic conditions for visual acuity and contrast sensitivity supports the hypothesis that the IOL is more pupillary-independent. Patient’ reported outcomes were good as evaluated with the Catquest 9-SF questionnaire. It remains to be confirmed that visual outcomes and patient satisfaction remain high with time. Further studies comparing the different trifocal IOLs, with a longer follow-up period would be necessary to better define the ideal IOL for each patient.

## Conclusion

The new Panoptix IOL provides good visual acuity outcomes in daily practice. The trifocal design does not appear to affect contrast sensitivity and visual function is similar in different lighting conditions, suggesting a low pupillary dependence. Patient reported outcomes reveal that spectacle requirement is low after bilateral implantation with a low incidence of glare and haloes. Therefore, it represents an option for patients who wish to be spectacle-free after cataract surgery with a good range of vision and a low rate of visual disturbances.

## Abbreviations

BCVA: Best-corrected visual acuity
cd: Candelas
cpd: Cycles per degree
D: Diopters
IOL: Intraocular lens
